# Nutrition labelling: a review of research on consumer and industry response in the global South

**DOI:** 10.3402/gha.v8.25912

**Published:** 2015-01-22

**Authors:** Jessie Mandle, Aviva Tugendhaft, Julia Michalow, Karen Hofman

**Affiliations:** PRICELESS SA, MRC/Wits Rural Public Health and Health Transitions Research Unit, University of the Witwatersrand, Johannesburg, South Africa

**Keywords:** nutrition label, global South, consumer behaviour, food industry, regulations and policy

## Abstract

**Background:**

To identify peer-reviewed research on consumers’ usage and attitudes towards the nutrition label and the food industry's response to labelling regulations outside Europe, North America, and Australia and to determine knowledge gaps for future research.

**Design:**

Narrative review.

**Results:**

This review identified nutrition labelling research from 20 countries in Asia, Africa, the Middle East, and Latin America. Consumers prefer that pre-packaged food include nutrition information, although there is a disparity between rates of use and comprehension. Consumer preference is for front-of-pack labelling and for information that shows per serving or portion as a reference unit, and label formats with graphics or symbols. Research on the food and beverage industry's response is more limited but shows that industry plays an active role in influencing legislation and regulation.

**Conclusions:**

Consumers around the world share preferences with consumers in higher income countries with respect to labelling. However, this may reflect the research study populations, who are often better educated than the general population. Investigation is required into how nutrition labels are received in emerging economies especially among the urban and rural poor, in order to assess the effectiveness of labelling policies. Further research into the outlook of the food and beverage industry, and also on expanded labelling regulations is a priority. Sharing context-specific research regarding labelling between countries in the global South could be mutually beneficial in evaluating obesity prevention policies and strategies.

Across the globe, rates of nutrition-related non-communicable diseases (NCDs) are on the rise (1). Once seen as a trend in wealthier countries, economically transitioning countries are now facing similar NCD burdens (2, 3). One factor driving the growing NCD burden is the increased consumption of cheap, energy dense, and nutrient poor foods. This so-called ‘Western’ diet is becoming more prevalent across the globe as more consumers eat pre-packaged foods and meals purchased outside of the home (2, 4). In response to increasing NCD rates, many governments are implementing multi-faceted policy interventions (5). One such policy is the adoption of nutrition labelling on pre-packaged foods and beverages. The Codex Alimentarius Commission, established by the Food and Agriculture Organization (FAO) and the World Health Organization (WHO), has developed standards for nutrition guidelines on food products (6). Many governments are revising their nutrition regulations as a means to not only meet food safety requirements but also as a government best practice for tackling nutrition-related NCDs (7, 8).

Labelling regulations have been adopted in many countries experiencing a population-wide ‘nutrition transition’ from traditional diets to contemporary patterns of food consumption (4, 8). Although the majority of labelling regulations exist in Europe, North America, Australia, and New Zealand (i.e. the global North), some countries in Asia, Africa, the Middle East, and Latin America (i.e. the global South) have also initiated regulations (Fig. 1).

**Fig. 1:**
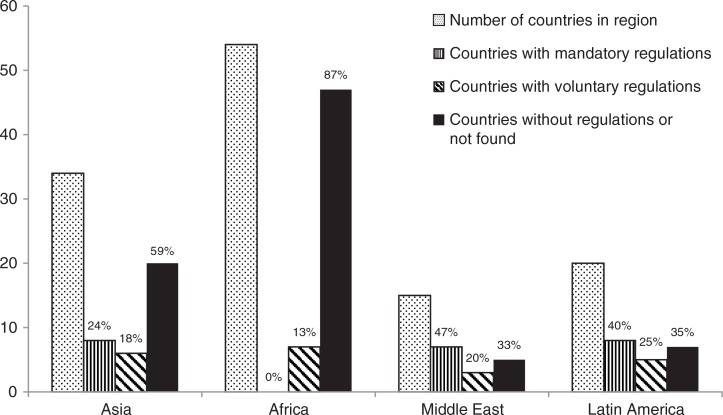
Food labelling regulations in the global South by region.

Nutrition label formats fall into two general categories: the back of package or BOP labels and the front of package or FOP labels. In 2014, BOP is the most prevalent label format worldwide (9) and at least 75% of the global population lives in countries with BOP labelling regulations (10). These regulations stipulate either mandatory labelling on all products or voluntary labelling for those foods that make certain health or nutritional claims. In 2012, the Codex Alimentarius Commission recommended mandatory nutrition guidelines even when health claims are not made on a product (6). The European Union Food Information Council shows that at least 44 countries outside the global North have mandatory or voluntary regulations (9).

FOP labels augment the BOP label information and provide consumers with interpretive symbols or logos to assess a product's overall nutrition. Label formats may include the Multiple Traffic Light system (MTL), Guideline Daily Amounts (GDAs), or nationally-endorsed health symbols, such as the ‘Choices’ logo system that meet certain nutritional criteria, providing a summary or ‘seal of approval’ on products (11). The majority of FOP labelling regulations that exist in the North are voluntary. Seven countries, Chile, Malaysia, Philippines, Singapore, South Africa, South Korea, and Thailand, in the global South are in the process of adopting some form of FOP labelling regulations (9, 12, 13).

Despite the increase in worldwide BOP and FOP labelling regulations, labelling research and reviews focus mainly on western countries with limited peer-reviewed analysis on labelling in countries in the global South (11, 14–22) . The need for more research evidence in these countries has been flagged as a priority (14, 18). This review seeks to identify under-represented research in the global South, and examines 1) consumer usage and attitudes towards nutrition FOP and BOP labelling and 2) the food industry's response to labelling regulations. An analysis of both groups draws attention to the state of labelling and reveals areas for future research in the global South.

## Methodology

Using a narrative review approach, we conducted a search of peer-reviewed literature on nutrition labelling in Asia, Africa, the Middle East, and Latin America. The narrative review approach was selected because it allows for investigation into an area under-represented in the literature and identifies areas for future investigation, yet would not meet the methodological criteria of a systematic review. Most of the articles reviewed are from low- and middle-income countries but also include countries that have transitioned to higher income brackets, that is, South Korea, Singapore, the United Arab Emirates, and Chile (23–29) .

We searched Google Scholar, Pubmed/Medline and Cochrane databases for any peer-reviewed articles in English, or English and another language published before June 2014. We searched for articles that contained at least one match with the search terms from three different sets: Set 1 terms were food, nutrition, nutritional, back of pack, front of pack; Set 2 terms were label, labelling, information, health logo, health symbol; Set 3 terms were the regions and countries from the global South. For example, a successful result might include an article that contained ‘nutrition’ and ‘label’ and ‘India’.

From Sets 1 to 3, we selected those articles that examined consumer responses to labelling, which included consumer knowledge, attitudes, behaviour, effectiveness or usage of the label. To identify articles on the food industry's response to labelling, we searched for articles that contained at least one match from Sets 1 to 3, as well industry-related terms: ‘food industry’, ‘beverage industry’, ‘regulation’ or ‘self-regulation’, ‘reformulation’, ‘harmonisation’, ‘lobby’, and ‘Big Food’. We selected articles that examined industry response to nutrition labelling, including industry's reaction or position concerning mandatory or voluntary nutrition labelling. See Table 1 for an overview of the narrative search.

**Table 1:** Overview of articles included in the search review

Response type	No. of studies (total: 34^a^)	Global South study setting (study number in parentheses)	Search terms
Consumer	27	Botswana (1), Chile (2), Ghana (2), India (3), Mexico (1), Lesotho (1), Malawi (1), Mauritius (1), Nigeria (1), Pakistan (1), Singapore (2), South Africa (6), South Korea (1), Sri Lanka (1), Trinidad (1), the United Arab Emirates (2)	Consumer knowledge, attitudes, behaviour, effectiveness, impact, usage in the general population and search term Sets 1–3
Consumer models	2	Middle- and low-income countries: Brazil (1), China (2), India (1), Israel (1), Mexico (1), Russia (1), South Africa (2)	
Industry	7	Brazil (1), Chile, Mexico (1), Morocco and Tunisia (1), Singapore (1), South Africa (1), Thailand (1)	Food industry, beverage industry, business, self-regulation, voluntary regulation, harmonisation, reformulation, lobby, ‘Big Food’ and search term Sets 1–3

Set 1–3 search terms: 1: food, nutrition, nutritional, back of pack, front of pack; 2: Label, labelling, information, health logo, or health symbol; 3: Regions and countries in Africa, Asia, Latin America and the Caribbean, the Middle East and Oceania.

a Two studies investigate consumer and industry response to labelling, Singapore (1) and Chile (1).

Given the limited data on these topics, we reviewed all articles in peer-reviewed journals, relying on the process of peer review to determine the rigour of articles selected; we did not use additional criteria to critically assess an article's validity. Articles were excluded, however, if they focused on specific groups or populations, such as the response of female shoppers or adolescents to labelling; this allowed us to investigate perspectives on nutrition labelling among the general population. In addition, because Google Scholar produces a wider selection of search results with diminishing relevance, we limited our review in Google to the first 50 results. Finally, after initial searches were completed, we then conducted forward searches from the references and from articles citing relevant search results.

## Results

We identified and reviewed articles from 20 countries in the global South. We identified 27 articles that investigate consumer usage and attitudes towards BOP or FOP nutrition labelling: South Korea (*n*=1), Singapore (*n*=2), India (*n*=3), Pakistan (*n*=1), Sri Lanka (*n*=1), United Emirates (*n*=2), Chile (*n*=2), Mexico (*n*=1), Trinidad (*n*=1), Botswana (*n*=1), Ghana (*n*=2), Lesotho (*n*=1), Malawi (*n*=1), Mauritius (*n*=1), Nigeria (*n*=1), and South Africa (*n*=6) (24–49). We also identified two articles that used modelling approaches to evaluate consumer responses to nutrition labelling (50, 51). Two articles addressed both consumer and industry responses to nutrition labelling (24, 28). Literature on the food industry's response to labelling, however, was more limited. We identified seven articles, which were often part of wider reviews or accounts of the food industry or national obesity policies (52). Furthermore, data on the food industry's reaction towards labelling primarily pertain to FOP labelling regulations. Articles came from the following countries: Chile (*n*=1), Mexico (*n*=1), Singapore (*n*=1), Thailand (*n*=1), South Africa (*n*=1), Brazil (*n*=1), and Morocco and Tunisia (*n*=1) (24, 28, 53–57). See Tables 2 and 3 for an overview of labelling regulations in the countries where research was identified (8, 9, 12, 13).

**Table 2:** Overview of labelling regulations (back of package) in countries with identified research

Back of package label regulations		

Mandatory	Voluntary	No information found
Brazil Chile India Thailand The United Arab Emirates	Mexico Mauritius Morocco Nigeria South Africa (mandatory BOP regulations recently introduced) South Korea Singapore Tunisia	Botswana Ghana Lesotho Malawi Pakistan Sri Lanka Trinidad

**Table 3:** Overview of labelling regulations (front of package) in countries with identified research

Front of package label regulations		

Mandatory	Voluntary	Pending
Chile: Hexagon warning label on certain products Thailand: Mandatory GDA labelling on snack products; the text ‘eat less, exercise more’ on certain children's snack products	South Korea: Traffic light labelling on children's food products Singapore: Healthier Choice logo	South Africa (voluntary FOP traffic light labels)

## Consumer usage and attitudes towards nutrition labelling in the global South

### Demographic predictors of consumer label use

Several demographic factors were associated with consumer label use and comprehension: education or socio-economic status, gender, family or household size, age, urban location, and ethnicity. *Education* level is positively associated with label use in India (30–32) , the United Arab Emirates (26, 27), Mexico (35), Mauritius (43), and South Africa (44); *socio-economic status* is also positively associated with label use in Botswana (37). *Gende*r is a predictor of label use in studies from Malawi (41), Ghana (39), the United Arab Emirates (26, 27), India (30), Mexico (35), Korea (23), and Singapore (25). Women are more likely to be health conscious and/or do the household shopping, characteristics associated with label use; an exception being a study from India that found men are more likely to look at the label and do the household shopping (30). A study in Mauritius found significant label use among women but no association between gender and label understanding (43). *Family and household size* are positively associated with label use; consumers with larger families and those shopping for children are more likely to use the label (26, 35, 37, 43), although household size was not a determinant in Pakistan (33). *Age* is also significant; however, age groups varied by country. *Urban-dwelling consumers* are positively associated with label use or comprehension when compared to those from rural areas or smaller metropolitan areas (30, 41); in Malawi, over half of urban consumers look at the nutrition information, compared to 4.9% of rural consumers. *Ethnicity* is also a predictor of label use; Malay consumers in Singapore had higher rates of use and White consumers in South Africa had greater label comprehension (25, 44).

### Behavioural determinants of label use

Consumers prefer that pre-packaged foods include nutrition information. In Sri Lanka, consumers called the nutrition label a ‘vital’ piece of information (34). There are certain behavioural traits that motivate label use across studies. Label users are more health conscious, aware of a health-diet link or have nutritional concerns (27, 34, 46, 49). Nutritional concerns may include specific dietary needs, weight control, or a disease diagnosis.

Additional factors include comparing products or purchasing a product for the first time, as seen in Botswana (37), Trinidad (36), India (31), Pakistan (33), and Mauritius (43). Consumers cite that they were looking for certain nutrition information, such as sugar, fats, calories, salt, or cholesterol. Different consumers look for different types of nutrients, varying across country and population. For example, in Malawi, urban consumers were more concerned about products with fat, salt, and sugar, while rural consumers sought products with vitamins and minerals, particularly vitamin A, iron and iodine (41).

Among the articles reviewed, the majority of consumers cited similar reasons for not using the nutrition label, despite differences in their demographic or geographic background. Reasons why consumers do not use the nutrition label information include lack of interest, time, and difficulties in understanding the label. Consumers report that the label is confusing in its terminology or language, and have a hard time locating the nutrition information. Consumers state that it takes too much time to read the label or it is even an ‘annoyance’ (23). Concerns that the label is not credible or that information provided is ‘dubious’ may also dissuade consumer use (35, 41).

Moreover, consumers prioritise other product qualities over the nutrition information. Consumers cite price, taste, appearance, brand, and overall familiarity with a product as reasons why they may disregard the label. Consumers look first at other label information, such as the expiration date, manufacturer details, food safety/storage information, and dietary information such as vegetarian or halal symbols (33, 38–40, 44, 48).

### The effectiveness of label use

The research on how labelling influences consumers can be divided into four measures of effectiveness: 1) self-reported use; 2) label comprehension as measured through self-reports or objective tests that gauge ‘actual’ understanding; 3) retail data to track how nutrition labels influence consumer purchasing; and 4) changes in consumer dietary intake or consumption patterns, as measured by longitudinal data or modelling approaches. The majority of the literature examines self-reported data or objective tests, with limited research on retail or population consumption.

#### Prevalence of self-reported use

Definitions of self-reported label use varied. The majority of studies ranged between 40 and 70% label use among the general population: 40.5% in Lesotho (40), 48% in South Africa (49), 55% in Chile (29), 58.5% in Trinidad (36), and 63.2% in the United Arab Emirates ‘sometimes to always’ read the nutrition facts panel (26). When asked if the label influences purchasing decisions, self-reports varied further: 65.8% ‘sometimes to always’ consider the label in Korea (23); 80.8% use the label in Nigeria (42), and 17% in Mexico (35). When purchasing a product for the first time, 11% use the nutrition label in India (31), 42.3% in Mauritius (43), and 70% in South Africa (47).

#### Consumer understanding

Rates of label comprehension are lower both in terms of consumer self-reports and objective measures of label literacy. In Malawi, 26.2% (41), 44% in Botswana (37), and 55.9% in Korea (23) report understanding the label. In Trinidad, 24.4% report reading but not understanding the label (36), a finding that confirms the challenges many consumers have with label comprehension.

Studies that evaluated consumers’ comprehension through objective tests found low rates of ‘actual’ understanding. Researchers in India, Mexico, Singapore, Chile, and South Africa all found levels of nutritional literacy to be lower than self-reported rates (25, 29, 31, 35, 44). In a Mexican study, 57% of consumers reported understanding the nutrition facts panel, yet only 1.2% of consumers surveyed correctly answered numerical information regarding the label (35).

In addition, consumers often experience difficulties determining the accuracy of manufacturer health or nutritional claims. Consumers note that they may rely on the manufacturers’ front of packaging claims as a main source of nutrition information [e.g. Mexico, the United Arab Emirates, South Africa (27, 35, 45)].

#### Retail data

We identified only one study that examines sales data from stores or vendors, providing information on the real-world purchasing trends of consumers. Sales data are used primarily to evaluate FOP labels, where fewer products carry the label. Sales data from stores in Singapore suggests that the voluntary FOP ‘Healthier Choices’ logo in Singapore may have a modest impact on food product sales and consumer demand (24).

#### Impact on dietary intake: longitudinal studies and modelling approaches

Studies examining the longitudinal impact of label use on diet or consumption patterns are also limited. The research from the same Singapore study suggests that the ‘Healthier Choices’ logo may be associated with a healthier diet. Data from a 2-day dietary study conducted by the Health Promotions Board of Singapore in 2010 found that individuals who consumed ‘Healthier Choices’ products were half as likely to exceed the recommended intake of saturated fat and more than twice as likely to meet dietary recommendations for calcium as individuals who did not consume any Choices products (24).

Modelling is another approach to gauge the label's population-wide impact, although few studies model the effects of labelling in the global South. Cecchini et al. (50) examined the population-wide health impacts and cost effectiveness of several interventions, including nutrition labelling in South Africa, China, India, Mexico, Russia, and Brazil, using England as a comparator. In the model, nutrition labelling resulted in improved population health outcomes in all countries, as measured through predicted disability-adjusted life-years (DALYs) saved. In a separate study, the potential impact of the Choices logo on the dietary intake of populations in seven countries of varying income levels was examined. The model replaced food items typically consumed in each country's diet with items that met criteria for the Choices logo system. Overall, dietary improvements were found across all seven countries, including reductions in saturated fats and sodium and sugars consumed, suggesting that the Choices labelling system could have broad health impacts (51).

### Label format and consumer preference

Several studies examine the type of label format and content that consumers prefer, and which label system is more conducive to comprehension (23, 29, 30, 32, 35, 40, 44). With respect to consumers’ preference for the label reference unit: per portion or per serving size is preferable to servings listed per 100 g (23, 29, 31, 40). One study in India found ‘per serving size’ is a more effective way to communicate nutrition information, instead of ‘per 100 grams’. Among consumers, 81% were able to use nutrition information when given the serving size, while only 7% of consumers were able to identify nutrition information when using the per 100 g format (31). Similarly, a telephone interview conducted among Chilean consumers also identified a preference for portion or serving information, instead of a serving size per 100 g (29). Korean consumers noted a preference for per package or portion information (23). Serving sizes per 100 g, while useful for product comparison, are more challenging for consumers to extract nutrition information, as found in Lesotho (40).

Regarding the format for nutrition information, consumer preferences are similar across the studies reviewed. Consumer preference includes: simple and clear labels that are easy to see at-a-glance and that avoid technical information; symbols or pictorial messages; health warnings or an explanation of important nutrients; information that is large in size; nutrition or health information endorsed by government agencies to ensure credibility; and standardised or mandatory label information on all products (23, 27, 29, 32, 35, 36, 40, 44). Consumers have trouble with numerical information and percentages, preferring text in lieu of, or in addition to, numerical information. Language and literacy is also an important consideration in labelling, but varies across countries; in a South African study, consumers noted that information listed in multiple languages would improve the label, while in a Ghanaian study, consumers were not deterred when the label was in a language they could not understand (38, 44).

Studies that examine consumer preference for specific FOP label formats are minimal. Korean consumers expressed a preference for the traffic light label (23). In Chile, research conducted among 1,300 head-of-household women found that a white and black warning label octagon with the message ‘Excess of –’ had the best performance in terms of visibility, comprehension, and change in intention-to-buy even after adjusting for educational level. The Chilean study also demonstrated that in order to have some impact, the size of the warning message had to be at least 10% of the front surface of the package of the product (28).

## Industry response in the global South

### Industry compliance with label policies or regulations

There is mixed evidence on industry's response to labelling regulations; the food industry may express support for labelling, especially when part of a larger national strategy to address obesity (53, 55, 57). In Mexico, the food and beverage industry initially signed on to the policy recommendations of a national panel that included labelling, although it later withdrew support for the Choices label system (53). In Morocco, representatives from the agri-food industry expressed support for potential mandatory nutrition labelling requirements when interviewed for research purposes (9, 57). In other instances, the food industry opposed FOP labelling requirements (28, 54, 57). In Chile, the industry lobbied against a bill targeting food labelling and advertising (28). While the initial response to labelling may vary, when mandatory FOP legislation is introduced, the industry plays a significant role in influencing the outcome of the regulations. In Thailand, after the food industry disputed a proposal for traffic light labelling supported by academic and consumer groups, the Thai Food and Drug Administration enacted GDA labelling as a compromise (54). In the case of Mexico, Chile, and Thailand, the industry's proposed regulatory requirements differ from the recommendations of academic or national committees (53, 54).

### Industry-initiated FOP labelling systems

In response to the growing interest in FOP food labels, food companies may initiate their own voluntary labelling scheme or implement GDA labels (58). In Mexico, the food industry promoted GDA labels (53). In South Africa, several food companies have started independently to initiate front-of-pack GDA labelling. Tiger Brands, for example, is now using the GDA percentages on its packaging (55).

### Voluntary regulation

There is limited data on this topic, although several studies from the global North have examined the prevalence of FOP labels when regulations are voluntary (19, 59). Singapore reports growth on the adoption of the voluntary FOP ‘Healthier Choices’ logo; products that carry the ‘Healthier Choices’ logo grew at an average annual rate of 5%, with logo penetration across 75 product categories (24).

### Product reformulation

Labelling regulations may influence food and beverage companies to reformulate their products and alter the characteristics of existing items to support healthier diets (60). Although, research exists from the global North (22, 60), there is limited evidence on the impact of product reformulation from the global South (52). Monteiro and Cannon (56) discuss instances in Brazil where manufacturers introduce reformulated products that carry front of package claims advertised as healthier.

## Discussion

The trend towards revised label regulations is growing worldwide. In the global South, consumers prefer to have nutrition labelling on pre-packaged foods, although use and comprehension is low, often due to difficulties interpreting BOP information. Consumers prefer government-endorsed nutrition information that is clear, easily visible, standardised, and includes symbols or pictures: label qualities in line with FOP systems. This pattern of positive consumer attitudes and high rates of self-reported use, but lower rates of real-world use and comprehension, is seen worldwide (14, 18). The characteristics, attitudes, and behavioural determinants of label users in the South are similar to consumers in the North (15, 16, 18). However, consumers in the global South are likely to prioritise other information on the food label before nutrition information, such as expiration date, manufacturer information and storage information.

The similarities between consumers in the South and North may also be a result of the study populations or the demographic factors investigated. The majority of the studies interviewed consumers in a single urban area, while only a few studies draw from multiple metropolitan areas or urban-rural hybrid areas (32, 48, 49). Other factors that may influence label use, such as religion or disability, are not widely discussed in the literature reviewed. While, as many studies note, respondents tend to be more educated or have higher income or literacy levels than the general population (27, 31, 32, 35, 46, 47). This may result in outcomes that do not adequately reflect the behaviours or determining factors for much of the population in the global South. Therefore, we cannot generalise that the majority of consumers in the global South prefer to have nutrition information included on pre-packaged foods. Consumers with lower education or those experiencing food insecurity may express different preferences.

Finally, our review begins to draw parallels between industry's responses in disparate settings. Although the evidence is limited, several accounts suggest that industry involvement in FOP legislation in the global South may reduce the regulatory strength of the label policy, leading to a label that is smaller, less visible, or more difficult for consumers to interpret (28, 54). Industry opposition to FOP labelling may also prevent implementation of labelling legislation (53). These cases resemble the experiences of the European Union and Australia, for example, where industry-led lobbying and public messaging campaigns have hindered calls for mandatory FOP labelling (61, 62).

Other types of industry responses, such as self-regulation and product reformulation, are similar to those observed in higher income countries (54, 63–66) . In the United States, for example, grocery store chains introduced their own FOP label systems in advance of governmental regulations. Researchers in higher income countries have found that product reformulation may lead to food products with healthier compositions. In the global South, however, some view product reformulation as more complicated within the broader context of multinational food companies: while companies may offer slightly healthier food items, the overall proliferation of ultra-processed products is associated with rising NCD rates and the dismantling of traditional dietary patterns (67).

### Knowledge gaps in the global South

This narrative review reveals several linked knowledge gaps and recommended areas for future research:

First, more research is needed on the effectiveness of food label interventions to better understand how consumers across different demographics use labels in real-world settings and the long-term health outcomes of labelling. Analysis must extend beyond consumer interviews or questionnaires to include in-store observational studies and population-wide measures, such as retail data to evaluate the label's impact on consumer purchasing habits, longitudinal studies to assess health outcomes, and cost-benefit modelling of labelling interventions.

Second, there is inadequate research on the attitudes and usage of labelling among the urban and rural poor. As these groups will likely bear the burden of NCDs in the future, more information is needed on how the nutrition label may influence their diet or purchasing decisions. Further research should explore how issues of language, literacy, or numeracy affect comprehension; if the nutrition label can increase awareness of a health-diet link; the role of the label and perceived costs of a ‘healthy’ diet; and what additional information should accompany the label to reach those with less access to health services and chronic disease management. These, and other areas for future investigation, will provide a more complete picture of label comprehension and could influence labelling policy decisions.

Third, there is a need for more data and transparency on the role that the food and beverage industry plays in influencing the food environment in the global South. The food industry's response can significantly alter the outcome of labelling regulations but literature on this topic is sparse. As new labelling policies emerge in the global South, more case studies are needed in order to analyse industry's response. The food industry's response to related labelling regulations, such as trans fat labels or genetically modified food products may also provide insights that could inform obesity-related policies. In addition, investigation into the relationship between label regulations and product reformulation is increasingly important, as the evidence to date has only hinted at the positive or negative outcomes that lie ahead.

Finally, research can explore the impact of expanded labelling policies. Singapore has initiated a ‘Healthier Hawker Programme’ to address food sold in food stalls, which holds promise for reaching informal food settings (24). South Korea and Taiwan have implemented menu labelling in restaurant fast food chains (13). Researchers have also noted the success of grocery store shelf-labelling programmes (22), an intervention which may be transferable to countries such as South Africa where grocery store chains play an increasingly important role in the food environment (55). Finally, many countries are adopting regional regulations and requirements, seen in Southeast Asia and the Mercosur countries in Latin America; sub-Saharan Africa should also explore the potential health and economic benefits of regional label harmonisation (20, 68).

## Limitations

As a narrative review highlighting gaps in the literature and areas for further study, this article is subject to several limitations. Despite our best attempts, new evidence and new regulations are constantly emerging, and may exist in languages other than English. Research that addresses industry's response to labelling was difficult to identify and may suggest a bias in the existing literature. This review demonstrates some of the challenges of studying nutrition labelling outside of Europe, North America, and Australia. These difficulties range from limited data to the challenges of defining boundaries that are both geographic and socio-economic. We used the terms ‘global North’ and ‘global South’, because defining a country by GDP alone (as in the case of World Bank low and middle-income countries) does not sufficiently capture the countries outside of Europe, Australia, and North America. A final constraint is our use of the peer review method to determine the rigour and validity of the studies we used. We did not account for the range of article types and varying definitions or measures that would have allowed us to draw a more systematic comparison.

## Conclusion

This review provides a foundation for further research into nutrition labelling, and contributes to the current literature in two ways. To our knowledge, this is the first review of labelling research in the global South. Second, this review is unique in that we investigate the response of both consumers and industry to labelling and label regulations. An understanding of both groups is critical to determining the impact of nutrition labelling in these settings. Future studies, however, should expand the investigation beyond this dichotomy of consumers and industry, to include other key groups that influence and shape nutrition-related trends and policies. Consumer groups, nutrition scientists, and advocacy groups could all play important roles in the complex food and policy environment of the global South.

Finally, nutrition labelling is just one of several policies that governments can implement to reduce rates of obesity and nutrition-related NCDs. To assess the true costs of labelling, more research is needed in several areas, including expanded labelling, as new policies are developing in this area. Governments need both country-specific evidence and international comparisons in order to adopt cost-effective obesity prevention strategies. The experiences of countries in the global South can provide important lessons and opportunities for different demographics in high-income settings.
